# Modern technologies for improving cleaning and disinfection of environmental surfaces in hospitals

**DOI:** 10.1186/s13756-016-0111-x

**Published:** 2016-04-11

**Authors:** John M. Boyce

**Affiliations:** J.M. Boyce Consulting, LLC, 62 Sonoma Lane, Middletown, CT 06457 USA

**Keywords:** Disinfection, Disinfectants, Cleaning, Ultraviolet light, UV-C, Hydrogen peroxide vapor

## Abstract

Experts agree that careful cleaning and disinfection of environmental surfaces are essential elements of effective infection prevention programs. However, traditional manual cleaning and disinfection practices in hospitals are often suboptimal. This is often due in part to a variety of personnel issues that many Environmental Services departments encounter. Failure to follow manufacturer’s recommendations for disinfectant use and lack of antimicrobial activity of some disinfectants against healthcare-associated pathogens may also affect the efficacy of disinfection practices.

Improved hydrogen peroxide-based liquid surface disinfectants and a combination product containing peracetic acid and hydrogen peroxide are effective alternatives to disinfectants currently in widespread use, and electrolyzed water (hypochlorous acid) and cold atmospheric pressure plasma show potential for use in hospitals. Creating “self-disinfecting” surfaces by coating medical equipment with metals such as copper or silver, or applying liquid compounds that have persistent antimicrobial activity surfaces are additional strategies that require further investigation.

Newer “no-touch” (automated) decontamination technologies include aerosol and vaporized hydrogen peroxide, mobile devices that emit continuous ultraviolet (UV-C) light, a pulsed-xenon UV light system, and use of high-intensity narrow-spectrum (405 nm) light. These “no-touch” technologies have been shown to reduce bacterial contamination of surfaces. A micro-condensation hydrogen peroxide system has been associated in multiple studies with reductions in healthcare-associated colonization or infection, while there is more limited evidence of infection reduction by the pulsed-xenon system. A recently completed prospective, randomized controlled trial of continuous UV-C light should help determine the extent to which this technology can reduce healthcare-associated colonization and infections.

In conclusion, continued efforts to improve traditional manual disinfection of surfaces are needed. In addition, Environmental Services departments should consider the use of newer disinfectants and no-touch decontamination technologies to improve disinfection of surfaces in healthcare.

## Background

In recent years, there is an increasing consensus that improved cleaning and disinfection of environmental surfaces is needed in healthcare facilities [1–4]. Experts generally agree on a number of areas, including the fact that careful cleaning and/or disinfection of environmental surfaces, daily and at time of patient discharge, are essential elements of effective infection prevention programs. Moreover, when disinfectants are used, they must be used appropriately to achieve the desired effects. However, there are a number of areas of disagreement and controversy regarding best practices for cleaning and disinfection of environmental surfaces. Some experts favor physical removal of microorganisms using only a detergent solution [3]. Other individuals believe that manual disinfection of surfaces using currently available disinfectants is adequate, and that newer approaches to disinfection are not necessary.

The purpose of this article is to summarize the many factors that affect standard cleaning and disinfection practices and to discuss modern technologies that can supplement traditional cleaning and disinfection methods.

### Personnel-related issues

Multiple studies have shown that manual cleaning and disinfection of surfaces in hospitals is suboptimal. In many facilities, only 40 to 50 % of surfaces that should be cleaned are wiped by housekeepers [5]. In addition, observational methods combined with use of adenosine triphosphate (ATP) bioluminescence have shown that individual housekeeper performance varies considerably [6]. One study found variations among housekeepers in the amount of time spent cleaning surfaces, the number of wipes used in each room, and the level of cleanliness achieved [6]. Specialized cleaning teams that included infection control personnel have been shown to reduce *C. difficile* surface contamination more effectively than routine housekeepers [7]. Personnel turnover among Environmental Services departments is a significant problem [8, 9], which may reach 30 to 50 % in some facilities. As a result, shortages in Environmental Services personnel were reported by more than 50 % of hospitals in a recent survey conducted in the United States [10]. Among housekeepers and nursing personnel, there is often confusion about who is responsible for cleaning various surfaces and equipment [11, 12].

### Issues related to disinfection protocols and practices

In addition to the above personnel-related issues, there are many other factors that can potentially have adverse effects on the efficacy of traditional cleaning and disinfection practices. The type of surface being cleaned or disinfected can affect the completeness with which bacteria are removed. For example, Ali et al. found that the type of material from which bed rails were made affected how well they could be cleaned by microfiber cloths, and that bacteria were removed more effectively by antibacterial wipes than by microfiber [13]. Disinfectants may be applied using inadequate contact times. Failure of housekeepers to use an adequate number of wipes per room can result in poor cleaning of surfaces [6]. Use of wipes without sufficient antimicrobial activity against target pathogens can result in poor disinfection of surfaces and can lead to spread of pathogens from one surface to another [14, 15]. Binding of quaternary ammonium disinfectants to cloths made of cotton or wipes containing substantial amounts of cellulose may reduce the antimicrobial efficacy of the disinfectant [16, 17]. At least one laboratory-based study has shown that detergent wipes have variable ability to remove pathogens from surfaces, and may in fact transfer pathogens between surfaces [18].

Inappropriate over-dilution of disinfectant solutions by housekeepers or by malfunctioning automated dilution systems may result in applying disinfectants using inappropriately low concentrations [9, 17]. For example, an investigation of housekeeping practices at a large teaching hospital included an audit of 33 automated disinfectant dispensing stations that mix concentrated disinfectant with water to yield a desired in-use quaternary ammonium concentration of 800 ppm [17]. Quaternary ammonium concentrations of solutions dispensed were tested using commercially-available test strips. The audit revealed that several dispensing stations yielded solutions with less than 200 ppm, approximately 50 % of stations delivered solutions with 200 to 400 ppm. An investigation revealed several flaws in the dispensing system. Inexpensive test strips and more complicated titration kits are available to monitor quaternary ammonium concentrations of disinfectants.

**Fig. 1:**
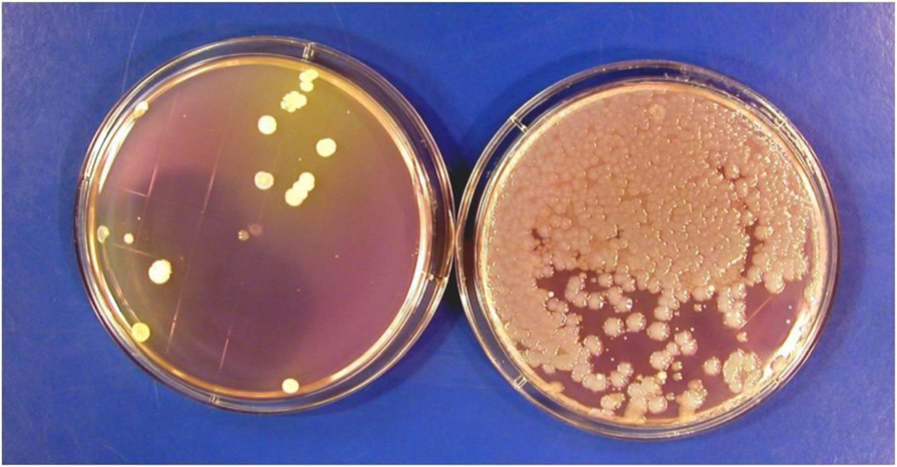
Contact agar plate cultures showing bacterial colonies recovered from a patient’s overbed table before (*left*) and after (*right*) the surface was cleaned by a housekeeper using contaminated quaternary ammonium disinfectant. Colonies on right are *Serratia marcescens* and *Achromobacter xylosoxidans*

Contamination of disinfectant solutions can occur, particularly if recommendations for their use are not followed [19–21]. For example, Kampf et al. recently reported that 28 buckets from 9 hospitals contained surface-active disinfectants (e.g., quarternary ammomium solutions) that were contaminated with *Achromobacter* or *Serratia* strains [21]. Buckets and roles of wipes had not been handled according to manufacturer recommendations. In studies that involved culturing high-touch surfaces in patient rooms before and shortly after housekeepers had performed routine cleaning, we found that cultures obtained from several surfaces in one room after cleaning yielded large numbers of *Serratia* and smaller numbers of *Achromobacter* which were not present before cleaning [Fig. 1] [20]. The housekeeper’s bucket of quaternary ammonium-based disinfectant contained 9.3 × 10^4^ CFUs/ml of gram-negative bacilli (mostly *Serratia marcescens* and fewer numbers of *Achromobacter xylosoxidans*). Pulsed-field gel electrophoresis demonstrated that *Serratia* isolates recovered from the disinfectant were the same strains as those recovered from surfaces in the patient room. Genome sequencing of one of the *Serratia* strains by collaborating investigators revealed that it contained four different *qac* resistance genes that permitted the organism to grow and survive in the disinfectant (unpublished data). If disinfectant contamination is suspected, a sample of the product can be used to inoculate a broth medium or solid agar containing neutralizers effective against the active agent(s) in the disinfectant solution.

Numerous studies have found that standard manual cleaning or disinfection of surfaces can reduce, but often does not eliminate, important pathogens such as *C. difficile,* staphylococci including methicillin-resistant *Staphylococcus aureus* (MRSA), vancomycin-resistant enterococci (VRE), and multi-drug-resistant *Acinetobacter* [7, 22–28]. Failure to adequately disinfect patient rooms at the time of hospital discharge contributes to the increased risk of acquisition of resistant pathogens among patients admitted to a room where the prior room occupant was colonized or infected with a multidrug-resistant pathogen [29–31].

### Monitoring housekeeping practices

**Fig. 2:**
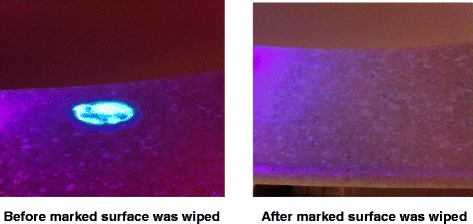
Photographs of a fluorescent marker visible with a “black light” on a high touch surface before cleaning (*left*), and absence of the fluorescent marker after cleaning was performed (*right*)

**Fig. 3:**
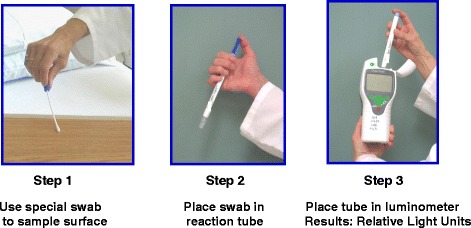
Three steps of an ATP bioluminescence assay for monitoring cleanliness of surfaces. Step 1: a special swab is used to sample the surface. Step 2: the swab is placed in a reaction tube and shaken for 10–15 s. Step 3: the reaction tube is placed in a luminometer and a result is reported as relative light units (RLUs). The higher the RLU value, the greater the amount of ATP detected on the surface

In order to improve standard cleaning and disinfection practices, it is recommended that the practices of housekeepers be monitored and that they receive feedback regarding their performance. However, monitoring of housekeeper performance is often not performed as frequently as needed, if at all [10]. Recently, fluorescent marking systems (Fig. 2) and ATP bioluminescence assays (Fig. 3) have proven useful for evaluating cleaning practices and providing housekeepers with feedback [32, 33]. Unfortunately, such objective means of monitoring the adequacy of cleaning/disinfection practices are not routinely used in many facilities [10]. Perhaps the lack of monitoring of housekeepers is due in part to the fact that monitoring activities can be time-consuming and must be conducted on an ongoing basis in order to be effective [34].

Given the multitude of challenges to achieving and maintaining adequate cleaning and disinfection in healthcare facilities, there is a need to consider the use of modern technologies designed to improve disinfection of surfaces in hospitals. New technologies fall into several categories, including: (A) new liquid surface disinfectants, (B) improved methods for applying disinfectants, (C) self-disinfecting surfaces, (D) light-activated photosensitizers, and (E) no-touch (automated) technologies.

## New liquid disinfectants

New disinfectants that are currently available or under development include improved hydrogen peroxide liquid disinfectants, peracetic acid-hydrogen peroxide combination, electrolyzed water, cold atmospheric pressure plasma, and polymeric guanidine. Several improved hydrogen peroxide disinfectants have been shown to be effective one-step cleaner/disinfectant agents that significantly reduce bacterial levels on surfaces [35–38]. In one study, use of a product containing 0.5 % (weight/volume) improved hydrogen peroxide was associated with fewer healthcare-associated infections when compared to an existing cleaning product, although all potential confounding variables were not analyzed [38]. Improved hydrogen peroxide liquid disinfectants can also be used to reduce contamination by multidrug-resistant pathogens on soft surfaces such as bedside curtains [14, 39]. Several of the improved hydrogen peroxide disinfectants also have activity against norovirus surrogate viruses, although they are not as potent as sodium hypochlorite (bleach) solutions [40]. These newer disinfectants have Environmental Protection Agency (EPA) safety rating of category IV (housekeepers do not need to wear any personal protective equipment while using these products).

A new sporicidal disinfectant that contains both peracetic acid and hydrogen peroxide has been shown to reduce bacterial levels on surfaces to a greater degree than a quaternary ammonium disinfectant in one study, and reduced contamination by *C.difficile*, MRSA, and VRE as effectively as sodium hypochlorite in another study [41, 42]. The product has a smell similar to vinegar that may be of concern when it is initially introduced. The combination product gives hospitals a potential alternative to sodium hypochlorite when a sporicidal disinfectant is needed.

Electrolyzed water (hypochlorous acid) disinfectant is produced by passing current through a solution of water and salt [43–45]. This promising disinfectant was shown to reduce bacterial levels on surfaces near patients to greater degree than a quaternary ammonium disinfectant in one study [43]. In another study, an electrolyzed water disinfectant significantly reduced MRSA, VRE and *C. difficile* spores in in-vitro experiments, and significantly reduced aerobic bacteria and *C. difficile* spores when sprayed onto medical equipment [44]. Spraying equipment was simple, required only approximately 15 s per application, and could be left to dry without wiping. One group of investigators found that electrolyzed water effectively reduced the number of aerobic bacteria (including staphylococci) on near-patient surfaces, but for reasons not well understood, appeared to allow regrowth of staphylococci within 24 h of application [45]. Further studies of this phenomenon are warranted. Electrolyzed water has the advantage of not leaving any toxic residues on surfaces. Issues related to stability of such products and logistic issues related to its use require additional study.

Cold-air atmospheric pressure plasma systems are being investigated for possible use as surface disinfectants in healthcare facilities [46–48]. In laboratory studies, the reactive oxygen species generated by these systems have bactericidal activity against a variety of pathogens, with variable activity against *C. difficile* spores [48]. Much more experience with cold-air atmospheric pressure plasma systems is needed to determine the practicality, efficacy and safety of using such systems in hospital settings. A novel nebulized solution of polymeric guanidine has been shown in one study to have antimicrobial activity against several healthcare-associated pathogens, and may warrant further investigation [49].

## New methods for applying disinfectants

Microfiber cloths or mops and ultramicrofiber cloths are among the relatively newer methods for applying liquid disinfectants to surfaces [50–54]. Some studies have shown increased cleaning efficacy of microfiber or ultramicrofiber cloths compared to standard cotton cloth or mops [51, 55]. However, it appears that all microfiber wipes are not equally effective [50]. Furthermore, if not used properly, there is some evidence that they may actually spread bacteria to other surfaces [53, 54]. When using microfiber cloths or mops, is important to know that the durability of these products is adversely affected by hypochlorite and high temperatures used during laundering and drying, and that their performance may decrease after multiple washings. One of the advantages of microfiber over cotton cloths is that microfiber is less likely than cotton cloths to bind quaternary ammonium disinfectants [16, 17]. However, presently, it is not clear how much the lower binding of microfiber cloths to quaternary ammonium disinfectants effects eradication of bacteria from contaminated surfaces. Additional studies are needed to better define the relative advantages and disadvantages of applying surface disinfectants with microfiber, cotton cloths and spunlace non-woven disposable wipes.

## Self-disinfecting surfaces

Creating “self-disinfecting surfaces” by coating surfaces with heavy metals such as copper or silver that have innate antimicrobial properties or applying to surfaces compounds that retain their antimicrobial activity for weeks or months has received some attention as a new strategy for disinfecting or preventing the growth of bacteria on surfaces in hospitals [56, 57]. Silver binds strongly with disulfide and sulfhydryl groups present in proteins of microbial cell walls, leading to cell death [56]. The antimicrobial activity of copper may be due primarily to its ability to form reactive oxygen radicals that damage nucleic acid and proteins [56]. Impregnating equipment surfaces with copper alloys has been shown to reduce bacterial contamination of surfaces [58–60], and in one study, coating several surfaces in hospital rooms with copper alloy was associated with reduction in incidence of HAIs [60]. Further studies of the long-term antimicrobial potency, practicality and cost-effectiveness of copper-coated surfaces are needed. Privacy curtains impregnated with silver have been shown to reduce or delay contamination of curtains with potential pathogens [61, 62].

Organosilane compounds are comprised of a surfactant plus an antimicrobial substance such as a quaternary ammonium moiety. These compounds are designed to minimize bacterial contamination of surfaces by maintaining their antimicrobial activity on surfaces for weeks or months. To date, the ability of these compounds to prevent contamination of surfaces for prolonged time periods is unclear. One study that applied compounds to surfaces using microfiber cloths failed to demonstrate continuing antimicrobial activity, where as two other studies using different application methods reported persistent antimicrobial activity of varying levels for differing time periods [63–65]. Further evaluation of organosilane-type compounds using a variety of application methods appears warranted. Polyhexamethylene biguanide disinfectant was found to reduce bacterial levels on surfaces for at least 24 h after application in one study [66].

## Light-activated photosensitizers

A few studies have explored the potential of applying of light-activated photosensitizers such as nanosized titanium dioxide to surfaces and using UV light to generate reactive oxygen species that can disinfect surfaces [67–70]. Activated titanium dioxide has been shown to have varying antimicrobial activity, with the relative susceptibility of agents against pathogens. Research on the use of light-activated photosensitizers is in early stages, and much more information about the feasibility and safety of using this strategy is needed.

## No-touch room decontamination methods

Examples of no-touch room decontamination technologies include: aerosolized hydrogen peroxide, hydrogen peroxide vapor systems, gaseous ozone, chlorine dioxide, saturated steam systems, peracetic acid/hydrogen peroxide fogging, mobile continuous ultraviolet devices, pulsed-xenon light devices, and high-intensity narrow-spectrum (405 nm) light [1, 3, 4, 71, 72].

### Aerosolized hydrogen peroxide

Aerosolized hydrogen peroxide systems that utilize 3 to 7 % hydrogen peroxide with or without the addition of silver ions have been evaluated by several investigators [25, 73–79]. Aerosols (which are not vapor) generally have particle sizes ranging from 2 to 12 μ, are injected into a room, followed by passive aeration. These systems have been shown to significantly reduce bacteria, generally a 4 log_10_ reduction of spores, although in several studies spores were not completely eradicated. One system has a sporicidal claim from the EPA in the United States. In one study, use of the aerosolized hydrogen peroxide system was associated with a reduction in *C. difficile* infection, and possible reduction of MRSA acquisition in a second study [25]. Like many other strategies in infection control, there are currently no randomized controlled trials of the efficacy of these systems in preventing health-care-associated infections.

### Hydrogen peroxide vapor

A “dry gas” vaporized hydrogen peroxide system that utilizes 30 % hydrogen peroxide has been shown to be effective against a variety of pathogens, including *Mycobacterium tuberculosis, Mycoplasma, Acinetobacter*, *C. difficile*, *Bacillus anthracis*, viruses, and prions [80–83]. In before/after studies, dry gas vaporized hydrogen peroxide system, when combined with other infection control measures, appears to have contributed to control of outbreaks of *Acinetobacter* in a long-term care facility and in two intensive care units in a hospital [84–86]. However, long cycle times have made it difficult to implement this system in healthcare facilities.

A micro-condensation hydrogen peroxide vapor system, which utilizes 35 % hydrogen peroxide, is effective in eradicating important pathogens including MRSA, VRE, *C. difficile, Klebsiella, Acinetobacter, Serratia, Mycobacterium tuberculosis*, fungi, and viruses. Laboratory-based and in-hospital studies documented significant reductions (often 10^6^ log_10_) of a number of these pathogens, with 92 to 100 % reduction of pathogens on surfaces [23, 83, 87–93]. In before/after trials, when used in conjunction with other measures, the micro-condensation hydrogen peroxide vapor system appears to have contributed to control of outbreaks caused by MRSA, multi drug-resistant Gram-negative bacteria, and *C. difficile* [78, 87, 94–99]. A prospective, controlled trial performed by Passaretti et al. demonstrated significant reduction in the risk of acquiring multidrug-resistant organisms (MDROs), especially VRE [30]. It has also been used to decontaminate the packaging of unused medical supplies removed from isolation rooms, instead of discarding such items [100]. This system has also been used to decontaminate rooms previously occupied by patients with the Lassa fever and Ebola virus infection [101, 102]. Despite the demonstrated ability of this system to eradicate nosocomial pathogens from surfaces, concerns over its cost and room turn-around-times have hampered adoption of this technology in healthcare settings. At least one study found that the micro-condensation hydrogen peroxide system can be implemented in hospitals when census levels are relatively high [103]. Recent improvements in the efficiency of the system permit more rapid turn-around-times than earlier equipment, which may lead to wider adoption. To date, there are no randomized, controlled trials establishing the impact of the micro-condensation hydrogen peroxide system on reduction of healthcare-associated infections. Other vapor- or aerosol-based no-touch disinfection technologies that have been described, but whose adoption appears to be limited include gaseous ozone, chlorine dioxide gas, and saturated steam systems [104–109].

### Ultraviolet light devices

Automated mobile ultraviolet light devices that continuously emit UV-C in the range of 254 nm can be placed in patient rooms after patient discharge and terminal cleaning has been performed. A number of these devices can be set to kill vegetative bacteria or to kill spores. These systems often reduce the VRE and MRSA by four or more log_10_, and *C. difficile* by 1–3 log_10_ [110–118]. In one comparative trial, a continuous UV-C light system resulted in lower log reductions than a micro-condensation hydrogen peroxide vapor system [119]. Advantages of the mobile, continuous UV-C light devices include their ease of use, minimal need for special training of environmental services personnel, and unlike hydrogen peroxide vapor systems, the ability to utilize the devices without having to seal room vents or doors. Recently, a prospective, multicenter randomized controlled trial comparing a mobile continuous UV-C light system with standard and other enhanced surface disinfection methods has been completed [120]. Results of the trial should be published in the near future.

A pulsed-xenon device, which does not use mercury bulbs to produce UV light, emits light in the 200–320 nm range. It has been shown to significantly reduce pathogens in patient rooms [121–127]. The manufacturer recommends placing device in 3 locations in a room with 5–7 min cycles (shorter than with some continuous UV-C systems). While a few studies utilizing the device reported reductions in *C. difficile* infection [122, 127], a more recent 8-month study in a large institution found no significant reduction in *C. difficile* infection rates hospital-wide or on four units with high *C. difficile* infection rates [128]. One carefully-performed trial which compared the pulsed-xenon system with a continuous UV-C light device found that log_10_ reductions of pathogens achieved with the pulsed-xenon system were lower than with the continuous UV-C light device [129]. Additional evaluation of the pulsed-xenon UV system by independent investigators is needed.

### High-intensity narrow-spectrum light

High-intensity narrow-spectrum (HINS) light, which is visible violet-blue light in the range of 405 nm has been tested as a means of disinfecting air and surfaces and hospital rooms. This technology targets intracellular porphyrins that absorb the light and produce reactive oxygen species [130–132]. Its antimicrobial efficacy is lower than UV-C light, but it can be used in areas occupied by patients. In one study, continuous HINS light showed a 27 to 75 % reduction in surface contamination by staphylococci compared to control areas [131]. Further investigation of this technology, including its level of activity against *C. difficile*, appears warranted.

### Photocatalytic disinfection

An enclosed air purifying system designed for use by NASA utilizes UV-activated titanium dioxide photocatalytic reactions to oxidize volatile organic compounds and airborne microorganisms. Since aerosolization of pathogens such as *S. aureus* and *C. difficile* during patient care activities is known to occur, there may be some interest in using such systems in patient rooms to reduce airborne bacteria may settle onto environmental surfaces [133].

Given the increasing interest in the above-mentioned new technologies for cleaning and disinfection of environmental surfaces, the Agency for Healthcare Research and Quality (AHRQ) recently commissioned an expert panel to review data regarding these modern technologies. The panel concluded that there is a relative lack of comparative studies addressing the relative effectiveness of various cleaning, disinfecting and monitoring strategies, and that future studies are needed that directly compare newer disinfecting and monitoring methods to one another and with traditional methods [4].

## Conclusions

In conclusion, manual cleaning and disinfection of environmental surfaces in healthcare facilities (daily and at patient discharge) are essential elements of infection prevention programs. Because many factors make it difficult to achieve high rates of effective disinfection on a routine and sustained basis, continued efforts to improve the quality and consistency of traditional cleaning and disinfection practices are needed. Given the many challenges in achieving desired levels of surface disinfection, adoption of modern technologies is indicated to supplement traditional methods. Further research into the efficacy and cost-effectiveness of newer technologies, and when to best apply them, is needed. As additional data become available, it is likely that newer liquid disinfectants and some no-touch room decontamination systems will be more widely adopted to supplement traditional cleaning and disinfection practices.
